# Can Scintimammography Help Differentiate the Nature of Suspected Masses Identified in Breast Ultrasound among Young Patients?

**DOI:** 10.1055/s-0045-1805044

**Published:** 2025-03-09

**Authors:** Esmaeil Gharepapagh, Neda Akhoundi, Ashraf Fakhari, Batool Seifi, Sonia Sedghian, Mahnaz Ranjkesh, Tohid Sarfaraz, Alireza Siami, Iman Yazdani Nia

**Affiliations:** 1 Department of Medical Radiation Sciences Research, Tabriz University of Medical Sciences, Tabriz, Iran; 2 Department of Radiology, Hillcrest Hospital, University of California San Diego, San Diego, California, United States; 3 Department of Radiology, Faculty of Medicine, Tabriz University of Medical Sciences, Tabriz, Iran; 4 Department of Anesthesiology, Tabriz University of Medical Sciences, Tabriz, Iran; 5 Department of Biostatistical Analyzer, Amirkabir University of Technology, Tehran, Iran; 6 Russell H. Morgan Department of Radiology and Radiological Sciences, Johns Hopkins Medicine, Baltimore, Maryland, United States

**Keywords:** scintimammography, ultrasound, biopsy

## Abstract

**Background:**

Breast cancer is the second leading cause of cancer deaths among women. Given the limitations of mammography in detecting breast cancer among young patients with suspected masses identified through ultrasound, our study aims to assess the effectiveness of scintimammography in distinguishing the nature of these masses.

**Methods:**

The study included 123 patients between the ages of 18 and 35, who were presented with breast masses categorized as Breast Imaging-Reporting and Data System III and IV based on ultrasound findings. A total of 134 breast masses were identified in the patients through ultrasound examination. Patients underwent radiopharmaceutical injection of 99mTc-MIBI (technetium-99m methoxyisobutylisonitrile) with a 15 to 20 mCi dose. The radiopharmaceutical uptake in the scans was assessed using a scoring system ranging from 0 to 3. Then, the scores were compared with biopsy results.

**Results:**

There was a statistically significant relationship between the absorption score reported by scintimammography and the pathological findings ( *p* = 0.001). The sensitivity and specificity of scintimammography in malignant masses considering cutoff point of 2 for absorption score were 96 and 92%, respectively.

**Conclusion:**

Based on the obtained results, scintimammography could be considered a diagnostic and complementary method to ultrasound in evaluating benign and malignant breast masses in young patients with dense breasts.

## Introduction

Breast cancer is the second leading cause of cancer deaths among women, with an increasing number of patients every year. 1 2 3 4 5 Early diagnosis of breast cancer is one of the best approaches to prevent complications of this disease. 6 The mammographic examination can detect masses of at least 2 mm. None of the physical examination and mammography methods can definitively confirm cancer. Therefore, a biopsy should be performed from the desired area. 7 8

Currently, mammography is the most used method for breast cancer screening. However, it has limitations, particularly in patients with dense breast tissue, often seen in younger patients. 9 10

Scintimammography could help as a complementary method in diagnosing suspected breast lesions and an initial screening for breast cancer, especially in cases of dense breast tissue and multifocal lesions. It can often reduce unnecessary biopsies significantly. 11 12 13 14 Scintimammography is a noninvasive functional radioisotope scan with a physiological rather than anatomical basis. It is analyzed by comparing the difference in radiopharmaceutical absorption of sestamibi by cancer cells and normal tissue. The difference in increased uptake between cells is due to increased vascularity, mitochondrial activity, and the degree of proliferation of cancer cells. Scintimammography appears unaffected by the anatomical changes seen following chemotherapy and radiotherapy, so this technique can be useful in monitoring the treatment of breast cancer patients. 15 16

Given the limitations of mammography in detecting breast cancer among young patients with suspected masses identified through ultrasound, the objective of our study was to evaluate the efficacy of scintimammography using technetium-99m methoxyisobutylisonitrile (99mTc-MIBI) in differentiating the characteristics of these masses to reduce the need for unnecessary biopsies in the future.

## Study Design

This cross-sectional study was conducted from May 2017 to September 2020 in the radiology department of a teaching hospital. A total of 123 patients were enrolled in the study. The inclusion criteria comprised individuals aged between 18 and 35 who presented with suspected breast masses classified as Breast Imaging-Reporting and Data System (BI-RADS) III or IV based on ultrasound findings. Exclusion criteria included pregnant patients, lactating patients, patients who had a mass biopsy in the last 2 weeks, and patients with a history of breast cancer undergoing chemotherapy and radiotherapy.

## Scintimammography Scan Methods

**Fig. 1:**
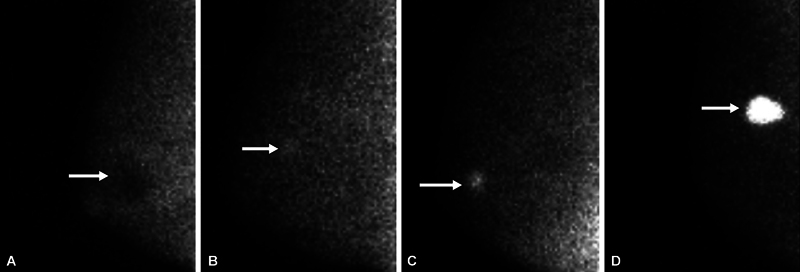
Examples of mass uptake intensity (referenced by Conners et al, 2012 17 ). ( **A** ) Mediolateral oblique molecular breast image in a 54-year-old woman shows photopenic mass (less intense than subcutaneous fat) ( *arrow* ). Ultrasound confirmed a simple cyst in this location (score 0). ( **B** ) Mediolateral oblique image in a 49-year-old woman shows mild intensity mass (equal to or slightly greater than subcutaneous fat) ( *arrow* ), shown to be fibroadenoma at biopsy (score 1). ( **C** ) Mediolateral oblique image in an 80-year-old woman shows a moderate-intensity mass (uptake visually greater than mild but less than twice as intense as subcutaneous fat) ( *arrow* ) due to grade II invasive ductal carcinoma (score 2). ( **D** ) Mediolateral oblique image in a 50-year-old woman shows marked intensity mass (at least twice as intense as subcutaneous fat) ( *arrow* ) due to grade III invasive ductal carcinoma (score 3).

Patients who met the inclusion criteria were referred to the nuclear medicine ward to perform a scintimammography scan with a high-resolution breast-specific gamma camera. Radiopharmaceutical injection of 99mTc-MIBI was performed with a dose of 15 to 20 mCi into the vein of the opposite breast arm (if bilateral evaluation of the breast is considered, a leg vein injection was performed). Then, the catheter route was washed with 10 mL of normal saline, static scans (5–10 minutes after injection) were performed in different views of the breast, and the resulting images were reported by a nuclear medicine specialist with 6 years of experience in this filed who was blinded to patients BI-RADS category, as follows:

1. Presence or absence of increased absorption in the breast area and scoring of absorption intensity according to the modified score of 0 to 3 by Conners et al, in which visual intensity of uptake in a lesion on gamma camera breast imaging can be characterized as photopenic (less intense than subcutaneous fat) scored as 0 ( Fig. 1A ), mild (equal or slightly greater than subcutaneous fat) scored as 1 ( Fig. 1B ), moderate (greater than mild, but less than twice as intense as subcutaneous fat) scored as 2 ( Fig. 1C ), and marked (at least twice as intense as subcutaneous fat) scored as 3 ( Fig. 1D ). 17
2. The possibility of artifacts (especially in the axillary and nipple area).

Finally, cases with indication were referred to biopsy of the mass, and the pathological results were compared with ultrasound and scintimammography findings. 18

### Statistical Analyses

Qualitative variables were expressed as frequency and percentage, and quantitative variables were expressed as mean values with standard deviation. The Kolmogorov–Smirnov test was used for a normal distribution of continuous variables. Sensitivity, specificity, positive and negative predictive value, and positive and negative predictive value ratios were calculated to verify the accuracy of the diagnostic test against the golden standard. Data analysis was performed using SPSS version 24 (SPSS Inc., Chicago, Illinois, United States).

## Results

This cross-sectional study studied 123 patients and 134 suspected breast masses. The mean standard deviation of the patients' ages was 31.06 ± 10.04 years, with a median of 24 years. The minimum age of patients was 18, and the maximum was 35. Most patients were referred to the relevant physician with a complaint of breast masses ( Tables 1 and 2 ).

**Table 1:** Frequency of signs and symptoms in patients with a breast mass

Signs and symptoms	Frequency	Percentage
Axillary mass	10	7.40
Breast mass	76	57
Nipple retraction	11	8.20
Nipple discharge	10	7.40
Breast tenderness	27	20

**Table 2:** Frequency and mean age of patients based on their BI-RADS category

BI-RADS category	Frequency	Percentage	Age (mean ± SD)
III	49	36.5	21.6 ± 10.31
IVa	59	44	32.5 ± 10.5
IVb	18	13.4	33.32 ± 9.4
IVc	8	5.9	31 ± 10.4
Total	134	100	31.06 ± 10.04

Abbreviations: BI-RADS, Breast Imaging-Reporting and Data System; SD, standard deviation.

Five (8%) and one (1%) cases with fibroadenoma showed moderate (score: 2) and high (score: 3) absorption, respectively. Forty-two cases (32%) had other benign pathology, of which 8 (66%) had a score of 0 and 2 (28%) were scored as 1 in scintimammography.

Ninety-six percent of malignant masses had a moderate to marked radiopharmaceutical absorption in scintimammography (8 cases with a score of 2 and 24 cases with a score of 3). There was a statistically significant relationship between scores 2 and 3 reported by scintimammography and the outcome of malignant pathology ( *p* = 0.001) ( Table 3 ).

**Table 3:** Comparison of absorption score in scintimammography with pathological results of breast masses

Pathological results Absorption Score	Malignant	Fibroadenoma	Other benign and cystic lesions	Total
0	0 (0%)	10 (16%)	28 (66%)	38 (28%)
1	1 (3%)	43 (72%)	12 (28%)	56 (41%)
2	8 (24%)	5 (8%)	1 (2%)	14 (10%)
3	24 (72%)	1 (1%)	1 (2%)	26 (19%)
Total	33 (25%)	59 (44%)	42 (32%)	134 (100%)

The diagnostic value of the scintimammography test was evaluated based on the biopsy results, which served as the gold standard for diagnosing malignant and benign masses. The receiver operating characteristic test was utilized, and the results are presented in Table 4 . The area under the curve was determined to be 0.798, with a *p* -value of 0.020, indicating statistical significance.

**Table 4:** Diagnostic value of scintimammography in breast masses considering the cutoff point of 2

			Pathological results	Total	Sensitivity	Specificity	PPV	NPV	Accuracy
		Positive	Negative						
**Absorption score** **≥ 2**	Positive	32	8	40	96%	92%	80%	99%	93%
	Negative	1	93	94					
	Total	33	101						

Abbreviations: NPV, negative predictive value; PPV, positive predictive value.

Based on our study results, a cutoff point of 2 was established for the absorption scores. Masses with scores below 2 were classified as benign, while scores above 2 indicated malignant absorption. These findings are summarized in Table 4 .

## Discussion

**Fig. 2:**
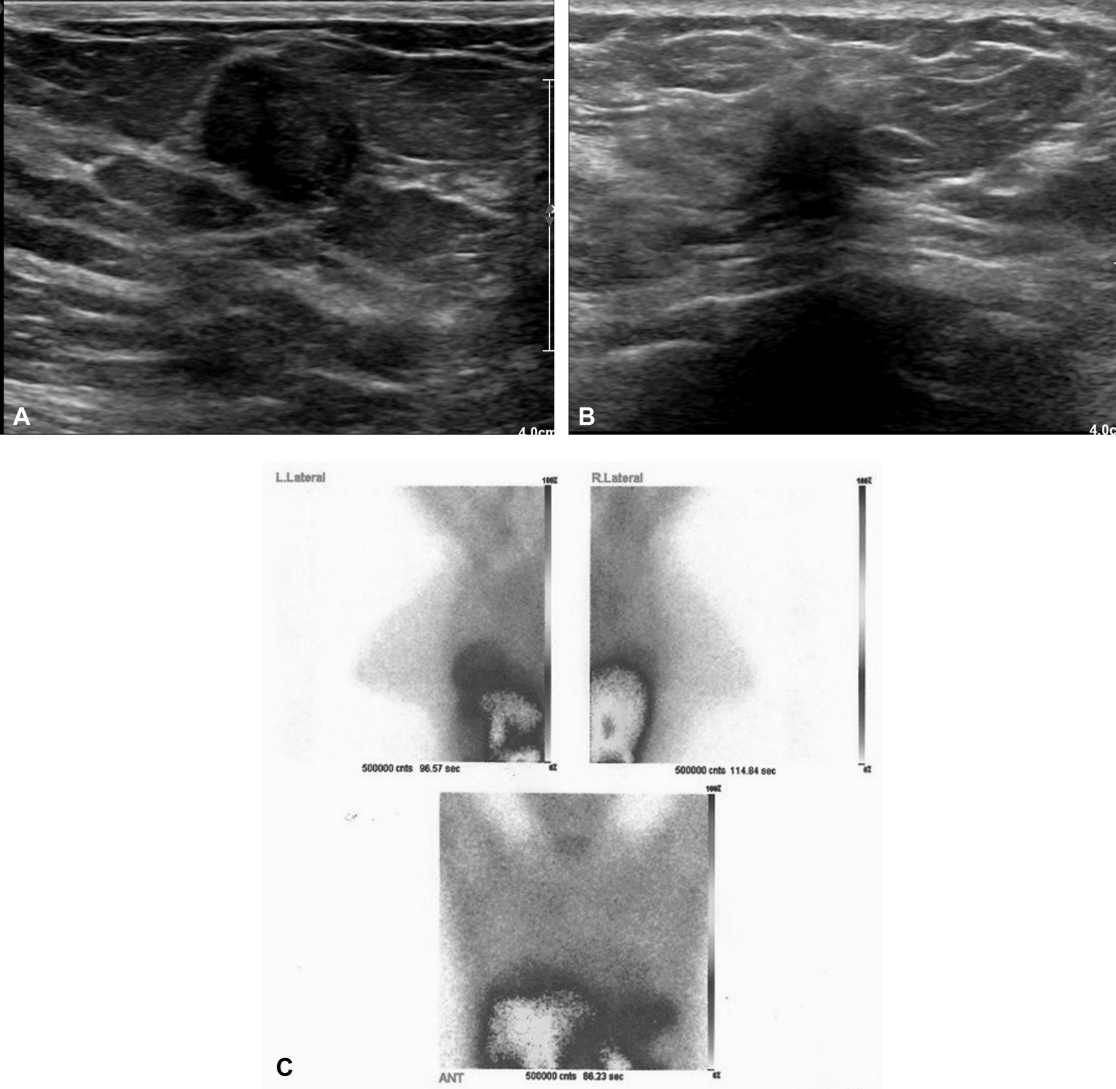
Two breast masses in a 29-year-old woman. ( **A** ) The ultrasound image of the right breast demonstrates a nearly well-circumscribed hypoechoic mass in near zone. ( **B** ) The ultrasound image of the left breast exhibits the spiculated hypoechoic mass with irregular borders in far zone of left breast. ( **C** ) The mediolateral oblique image shows marked intensity mass due to invasive ductal carcinoma (score 3) in the left breast and mild intensity mass (equal to or slightly greater than subcutaneous fat), shown to be fibroadenoma at biopsy (score 1) in right breast.

**Fig. 3:**
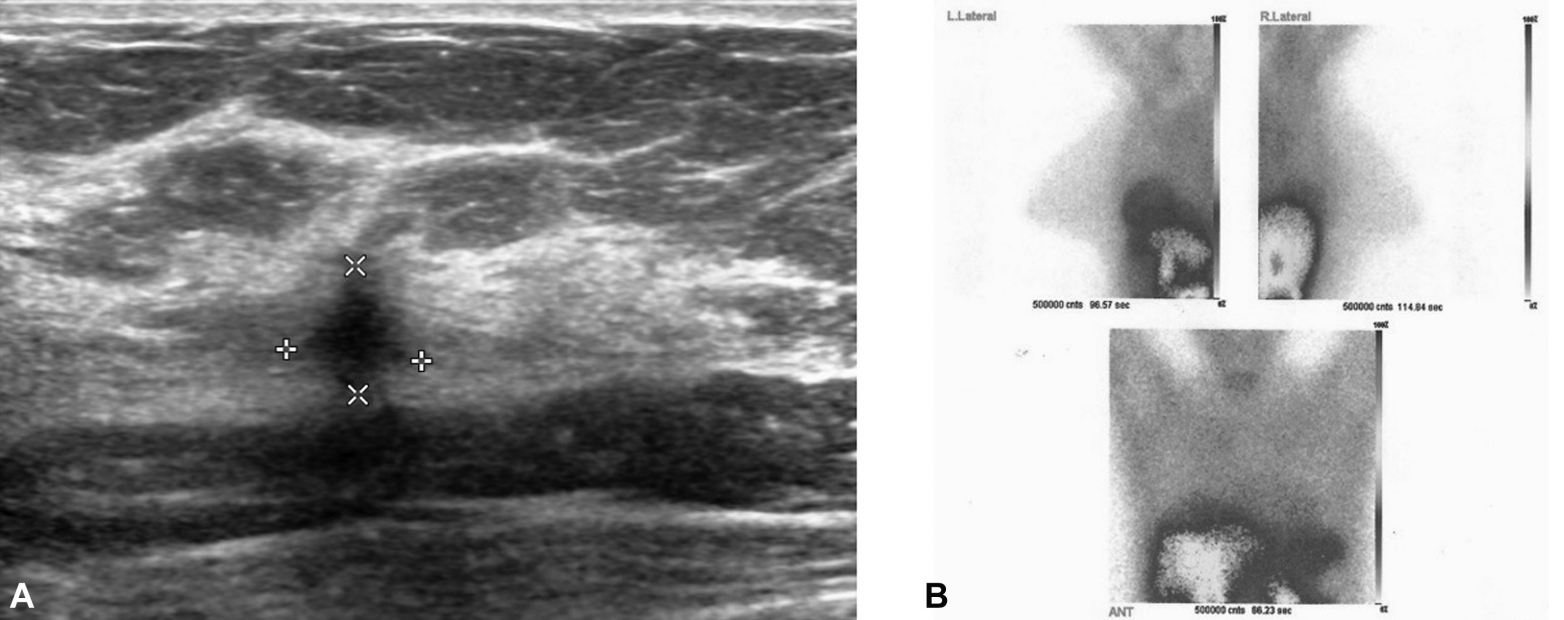
Left breast mass in a 31-year-old woman. ( **A** ) The ultrasound image of the left breast demonstrates a spiculated hypoechoic mass with irregular borders in far zone of left breast. ( **B** ) The mediolateral oblique image shows marked intensity mass due to invasive ductal carcinoma (score 3) in the left breast.

In recent years, 99mTc-MIBI breast scan has been used as a complementary imaging technique to increase the sensitivity and specificity of breast imaging. The primary objective of the present study was to evaluate the effectiveness of scintimammography using 99mTc-MIBI in differentiating the nature of suspicious masses identified through ultrasound, specifically those categorized as BI-RADS III and IV. Additionally, the study aimed to compare the scintimammography results with the findings obtained from biopsy procedures focusing on young patients ( Figs. 2 and 3 ). In the present study, the sensitivity, specificity, positive predictive value, and negative predictive value of scintimammography in malignant masses considering the cutoff point of 2 for absorption score were 96, 92, 80, and 99%, respectively. As mentioned, the results show the high power of scintimammography confirmation in benign breast masses and a complementary diagnostic aid method in malignant breast masses.

Studies conducted in recent years to evaluate breast cancer have shown high sensitivity and specificity for scintimammography, confirming our results. 11 18 19 20 21 The sensitivity and specificity of this method have been reported between 83 to 96% and 86 to 100% in the initial studies, respectively. 22 23 Our study also showed high sensitivity, specificity, accuracy, and negative predictive value for scintimammography. In a study conducted by Khalkhali et al 21 involving 637 breast lesions, the reported sensitivity and specificity were 75.4 and 82.7%, respectively. Indeed, the variation in sensitivity and specificity between the study conducted by them and our study could be attributed to differences in the population groups under investigation. This modified scoring system seems to improve the sensitivity and specificity of scintimammography in detecting the nature of breast masses. In the study of Sampalis et al, 24 it was reported that the use of scintimammography in comparison with mammography could help in the early detection of breast cancer in palpable and nonpalpable lesions. Also, this study reported a high negative predictive value (99%) for scintimammography, same as ours. On the other hand, in the study of Tiling et al, 25 it was reported that tumor size can also be considered a limiting variable in breast scintimammography. In some studies, the absorption pattern was a more accurate criterion than the amount of absorption in interpreting the scan. 26 27 This study is the first attempt to evaluate the diagnostic value of scintimammography in differentiating the nature of suspected masses based on their absorption score (0–1 to 2–3) and determine an optimal cutoff point for this purpose among young patients with BI-RADS III and IV classifications on ultrasound.

## Conclusion

According to the results obtained in this study, using a cutoff score above 2, scintimammography showed a diagnostic accuracy of 93% in identifying malignant masses. The sensitivity of scintimammography was determined to be 96%. These findings suggest that scintimammography has high sensitivity and can serve as a complementary method to ultrasound, potentially reducing the need for unnecessary negative biopsies in young individuals with dense breast tissue.
